# Refuting a Temporal Correlation: Interictal Epileptic Discharges Do Not Preferentially Occur During Respiratory Events in Patients With Sleep‐Related Breathing Disorder and Epilepsy

**DOI:** 10.1111/jsr.70021

**Published:** 2025-02-23

**Authors:** Christian M. Horvath, Hristina Drangova, Jakub Stefela, Carolin Schäfer, Frederic Zubler

**Affiliations:** ^1^ Department of Pulmonary Medicine, Allergology and Clinical Immunology, Bern University Hospital University of Bern Bern Switzerland; ^2^ Sleep‐Wake‐Epilepsy‐Center, Department of Neurology, Inselspital, Bern University Hospital University of Bern Bern Switzerland; ^3^ Department of Neurology, St. Anne's University Hospital and Faculty of Medicine Masaryk University Brno Czech Republic; ^4^ Department of Neurology, Spitalzentrum Biel University of Bern Biel/Bienne Switzerland

**Keywords:** apnea, epilepsy, interictal epileptiform discharge, polysomnography, sleep relating breathing disorder

## Abstract

The bidirectional interaction between sleep and epilepsy is well known. In particular, it has been established that sleep apnea can worsen epilepsy, whereas sleep apnea (SA) treatment has a beneficial effect on seizure control. However, the exact mechanisms whereby SA promotes epileptic seizures are unknown. We set out to examine whether interictal epileptic discharges (IED), one of the hallmarks of epilepsy, occur predominantly during respiratory events (RE, apnea or hypopnea) or desaturations in patients with obstructive SA (OSA) and epilepsy. Adult patients (> 18) who underwent a video‐polysomnography at the Bern University Hospital between 2012 and 2020 with an apnea‐hypopnea‐index (AHI) ≥ 10/h and IED were included in this retrospective study. IED density (per hour) was computed during and outside RE and oxygen desaturations (3%) using the AASM criteria and an extended definition. A total of 27 patients (9 females) met the inclusion criteria. The median age was 49 years and the median AHI was 17.4/h. There was no statistically significant difference in IED density in phases of sleep with RE compared to sleep without (median 3.6 [IQR 0.2–8.0] vs. 6.3 [3.7–19.7], *p* = 0.055). In the extended definition of RE, IED density was significantly lower during RE: 2.6 [0.3–6.6] versus 6.7 [3.9–20.5], *p* = 0.017. Desaturations were similarly associated with lower IED density in both analyses: 2.2 [0–7.4] versus 6.4 [3.4–18.4], *p* = 0.009 and 2.6 [0–6.7] versus 6.8 [3.4–18.5], *p* = 0.012. Our study shows that the influence of OSA on epileptic activity is probably indirect and does not result solely from immediate hypoxemia.

## 1 Introduction

The bidirectional influence of epilepsy and sleep has been recognised since antiquity (Nobili et al. 2022; Aldrich and Aldrich 1999). Already Hippocrates noted that some patients tend to experience seizure only during sleep (Souques 1936). Later, it was discovered that not only sleep, but also the different sleep and wake stages have an impact on the occurrence of seizures and interictal epileptic discharges (IED) (Nobili et al. 2021; Latreille et al. 2018; Campana et al. 2017). Moreover, epileptic seizures and IED can disrupt sleep and affect breathing control, indicating that individuals with epilepsy are more likely to suffer from sleep disorders (Nobili et al. 2022; Nobili et al. 2021; Latreille et al. 2018; Nishimura et al. 2015; Somboon et al. 2019). Conversely, sleep disorders are known to increase the susceptibility to epilepsy and trigger epileptic seizures (Latreille et al. 2018; Gibbs et al. 2016). The bidirectional relationship between epilepsy and sleep disorders has been particularly well established for obstructive sleep apnea (OSA) (Somboon et al. 2019; Lin et al. 2017; Goyal et al. 2023; Devinsky et al. 1994).

Epilepsy is a pathological condition characterised by a chronic predisposition to generate epileptic seizures (Fisher et al. 2005). The current classification of epilepsies is based on the type of seizures, in particular seizures with focal onset (when the seizure originates from a localised region in one hemisphere) or primarily generalised (the seizure originates in deeper subcortical circuits with seemingly simultaneous involvement of bilateral cortical structures) (Scheffer et al. 2017). In some persons with epilepsy, the probability of seizure occurrence is correlated with the IED density on a time scale of hours or days (Baud et al. 2018; Proix et al. 2021), whereas on a shorter scale (minutes to seconds) there is no systematic correlation, as some patients will have more, and other less IEDs just before a seizure (Baud et al. 2018; Karoly et al. 2021; Schroeder et al. 2023; Karoly et al. 2016). Also, the influence of sleep on ictal and interictal epileptic activity (and their appearance during EEG recordings) often depends on the type and aetiology of the epilepsy (Nobili et al. 2020) and on the stability of sleep (Manni et al. 2005; Parrino et al. 2000).

OSA is characterised by recurrent episodes of partial or complete airflow cessation in the upper airways during sleep, often leading to oxygen desaturation and arousals from sleep (Gottlieb and Punjabi 2020). This condition has been recognised as a significant health concern due to its potential impact on cardiovascular health, cognitive function, and overall quality of life. Sleep apnea may play a pivotal role in the exacerbation of seizures in individuals with epilepsy (Lin et al. 2017). Conversely, the treatment of sleep apnea may lead to better seizure control (Lin et al. 2017). However, the precise pathophysiological mechanisms underlying the effect of OSA on epileptic activity remain unclear (Goyal et al. 2023). It is not clear whether intermittent hypoxia—as seen in OSA—leads immediately to an increase of epileptic activity in the brain. A few case reports exist on patients in whom seizures or interictal epileptic activity were predominantly linked to obstructive or central apnea (Devinsky et al. 1994; Nadkarni et al. 2012; Nguyen‐Michel et al. 2015), but to the best of our knowledge no systematic study on this subject has been conducted. Here, we set out to investigate the temporal relationship between respiratory events and IED in a monocentric cohort over 8 years.

## 2 Methods

### 2.1 Patient Pre‐Selection

This retrospective study was authorised by the Ethic commission of the Canton of Bern (Protocol 2019–02370). We used the patient documentation system of the Sleep‐Wake‐Epilepsy‐Center of the Bern University Hospital to screen for patients who underwent a diagnostic video‐polysomnography (PSG) in routine clinical practice at the sleep laboratory of our institution between 08/2012 and 08/2020. Pre‐selection of potential candidates was based on the clinical report and consisted of: age ≥ 18 years; apnea‐hypopnea‐index (AHI) ≥ 10/h; reported epileptiform activity during the PSG (searching the following key words in the report: ‘spike(s)’, ‘spike–wave’, ‘sharp wave’, ‘sharp‐slow‐wave’, ‘epileptiform’, ‘seizure(s)’), a signed a consent allowing for the re‐use of clinical data for research purposes.

### 2.2 PSG Recording and Initial Interpretation

All PSG recordings were performed using a RemLogic (Embla Systems LLC) system with electroencephalography (EEG), electrooculography, submental electromyography, electrocardiography, respiratory flow, and videography. Depending on the clinical indication, EEG was recorded using 19 electrodes as specified in the international 10:20 system or only a subset of 6 electrodes (corresponding to positions F3, F4, C3, C4, O1, O2), plus electrodes A1 and A2 placed on the mastoid left and right respectively; a ground electrode was placed near the Fz region; the reference electrode was A1. EEG sampling rate was 200 Hz. In clinical routine, PSGs were scored manually on 30‐s epochs according to the 2012 Manual of the American Association of Sleep Medicine (AASM) by a somnologist or sleep‐laboratory technician, whereas the respiratory events were initially marked semi‐automatically and then reviewed for clinical interpretation by a resident and an attendant with specialisation in sleep medicine.

### 2.3 Scoring and Final Patient Inclusion

After pre‐selection, PSGs were reviewed in detail for this study both by a board‐certified pneumologist and sleep specialist (CH) and a board‐certified neurologist with additional certification in epileptology and electroencephalography (HD, JS, CS, FZ). We used the rules of the American Association of Sleep Medicine 2.6 for re‐scoring respiratory events (3% oxygen desaturation index threshold) (Berry and Abreu 2020). By contrast, we did not rescore the sleep stages and retained the original clinical sleep scoring done by sleep lab technicians. Interictal epileptiform discharges (IED) were marked visually using standard criteria (Kane et al. 2017). Discussion of borderline EEG transients with another colleague was left to the discretion of each neurologist. Sleep stage scoring, timing of arousal and IEDs, starting and stopping time of apnea and hypopnea were exported as text file using the RemLogic built‐in export function.

### 2.4 Analysis of IED Density

IED density was defined as the number of spikes or sharp waves per hour under a specific condition (e.g., the IED density during sleep without desaturation for a specific patient was computed as the total number of IED observed during sleep outside of desaturations divided by the total time of sleep outside desaturation expressed in hour). When considering IED density in apnea, hypopnea or desaturation, we performed the analysis twice: Once using the strict starting and stopping time of the event as scored, and once adding a time window of 5 s before the starting and 10 s after the stopping time of each event. This extended definition was motivated by the surge in spikes observed in certain type of epilepsies a few seconds before a cortical arousal (ca. 5 s before Peter‐Derex et al. 2020), and the occurrence of delayed arousal after apnea or hypopnea (6 to 15 s Zitting et al. 2023). Similarly, seizure density was defined as the number of seizures starting during a certain condition per hour of the said condition. Analysis was performed using Matlab R2017a (Version 9.2, Matworks, Natwick, MA) with custom made scripts.

### 2.5 Statistics

When comparing IED density between two conditions, statistical significance was assessed with a two‐tailed Wilcoxon signed rank test. As correction for multiple comparisons we used the Benjamini–Krieger–Yekutieli false discovery rate control (Groppe et al. 2011). When comparing IED density between sleep stages (5 conditions), we used a Kruskal–Wallis test; then, in case of significant difference (*p* < 0.05), a post hoc pairwise comparison was performed using a Mann–Whitney U‐test with Tukey's significant difference criterion (built‐in Matlab functions ‘kruskalwallis’ and ‘multcompare’).

## 3 Results

### 3.1 Demographics

**FIGURE 1:**
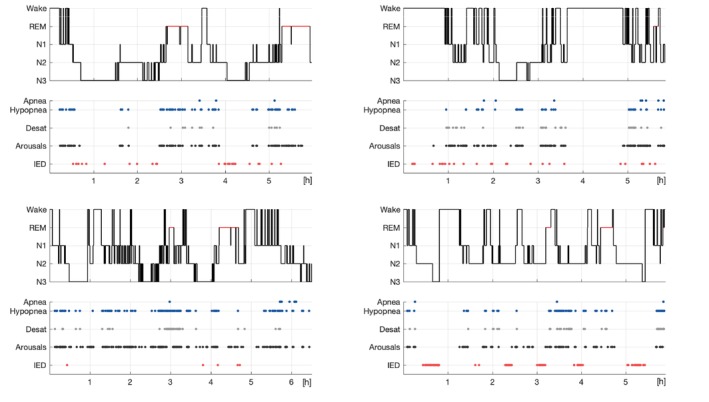
Representative examples. Summary of polysomnographic finding in four patients. For each patient we show the hypnogram together with the events analysed in the present study, namely respiratory events (apnea/hypopnea), desaturations, arousals and interictal epileptiform discharges (IED).

Fifty‐six patients were preselected based on screening the documentation system. Of those, 36 were later excluded because of AHI < 10 after rescoring and 3 because of absence of clear IED after detailed review of the EEG, resulting in the inclusion of 27 patients (9 females). The median age was 49 (quartile 1–3: [39–59]), the median AHI was 20.4 [13.8–25.1], all had OSA with very few central apneas. Patient demographics is summarised in Table 1, whereas detailed information about each patient is provided as Tables S1–S3. Examples of polysomnographic recordings are shown in Figure 1.

**TABLE 1:** Patient demographics and polysomnographic findings.

*N*	27
Age (years)	49 (39–59)
Female	9 (33%)
Prior diagnosis of epilepsy	24 (89%)
Type of epilepsy	
Structural/focal seizures	24 (89%)
Genetic/primarily generalised seizures	1 (4%)
Unknown	2 (7%)
Seizure suppressive medication	
None	4 (15%)
One	12 (44%)
Two	7 (26%)
Three	4 (15%)
Indication for PSG^a^	
Suspected sleep apnea	21 (78%)
Excessive daytime sleepiness	1 (4%)
Suspected sleep‐related epileptic activity	11 (41%)
10–20 EEG	23 (85%)
Total sleep time (min)	300 (243–330)
Sleep stage N1 (% total sleep time)	25 (18–38)
Sleep stage N2 (% total sleep time)	43 (35–51)
Sleep stage N3 (% total sleep time)	15 (5–23)
Sleep stage REM (% total sleep time)	12 (7–19)
Sleep efficiency (%)	83 (66–88)
AHI [h^−1^]	17.4 (14.1–25.1)
Central AI [h^−1^]	0.4 (0.0–0.7)
Obstructive AI [h^−1^]	2.1 (0.4–6.0)
REM AHI [h^−1^]	16.2 (11.5–27.8)
NREM AHI [h^−1^]	16.8 (12.6–25.8)
ODI 3% [h^−1^]	7.8 (5.8–17.9)
Mean SpO_2_ [%]	93.4 (91.8–94.8)
Total sleep time [%] with SpO_2_ < 90%	1.2 (0.2–4.1)

*Note*: Continuous values are given as median with quartile 1–3, binary values as number (percentage).

Abbreviations: AHI (apnea‐hypopnea index), AI (apnea index), EEG (electroencephalography), ODI (oxygen saturation index), REM (rapid eye movement), PSG (polysomnography), SpO_2_ (saturation of peripheral oxygen).

^a^ Non mutually exclusive.

### 3.2 IED Density and Sleep Stage

**FIGURE 2:**
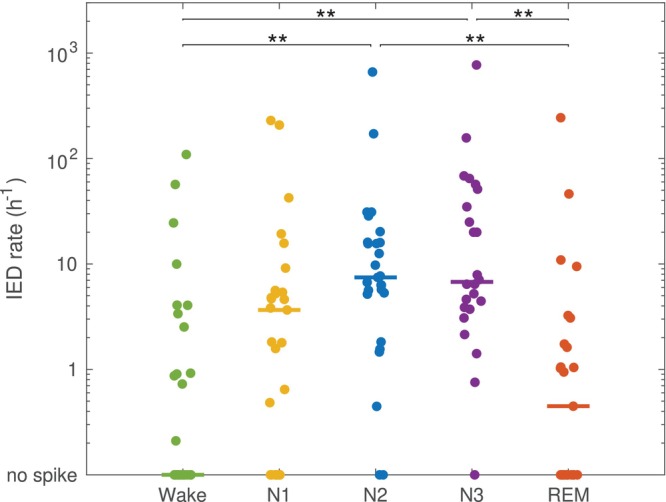
Density of interictal epileptiform discharges (IED) in different wake and sleep stages. The density was significantly higher in sleep stage N2 than in wakefulness and REM sleep, and also significantly higher in N3 than in wakefulness and REM. Horizontal bars represent the median. ** denotes a significant difference with *p* < 0.01.

First, we analysed the effect of sleep and wakefulness stages on the IED density. Results are shown in Figure 2. Of note, 3 patients had no N3 sleep and 2 patients had no REM sleep during the PSG night. A group level analysis indicated a significant difference of IED density between groups (*p* < 0.001). A post hoc multiple comparison showed that: (i) the IED density was significantly higher in N2 (median 7.5/h, quartile 1–3: [5.3–16.0]) than in wake (0, [0–2.1]) (*p* < 0.001) or in REM (0.4 [0–2.1]) (*p* < 0.001); (ii) the density in N3 (6.8, [3.7–43.0]) was also significantly higher than in wake (*p* < 0.001) or in REM (*p* < 0.001); (iii) The density in N1 (3.6 [0–5.5]) was not significantly different from any other sleep stage.

### 3.3 IED Density and Respiratory Events

**FIGURE 3:**
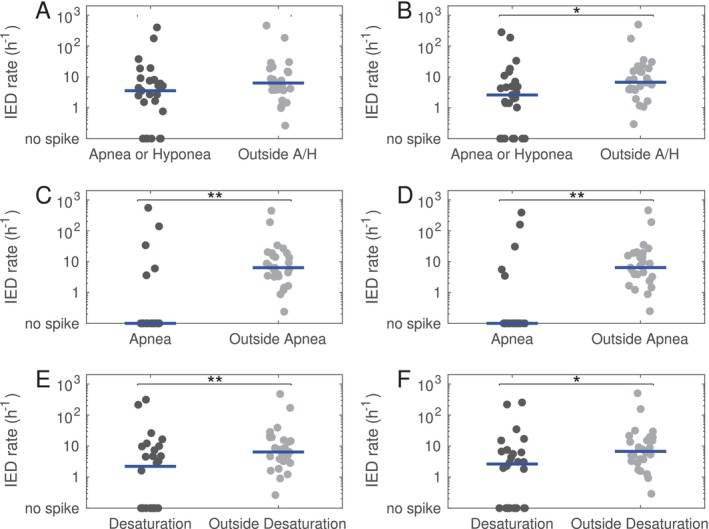
Density of interictal epileptiform discharges (IED) in sleep during and outside respiratory events. (A) The IED density tendentially lower in sleep during an apnea or a hypopnea than in sleep outside apnea or hypopnea, however the difference was not significant. (B) Using an extended definition (i.e., starting 5 s and stopping 10 s after the limits according to the AASM scoring rules), the reduction in IED during apnea or hypopnea became significant. (C) The IED was significantly lower during apnea than outside apnea. (D) The same result was found with the extended definition of apnea. (E) The IED density was significantly lower in sleep during a desaturation than in sleep outside desaturation. (F) The result was also significant for the extended definition of desaturation. (Horizontal bars represent the median. *p < 0.05, **p < 0.01).

Second, we investigated the influence of three different events, namely (i) respiratory events (RE, apnea and hypopnea), (ii) apnea alone, (iii) desaturation, on the IED density. To this end we compared the IED in sleep without and in sleep with respiratory events. We performed the same analysis twice. First, using the official definition of the AASM; and second, with addition of a time window of 5 s before and 10 s after the respiratory events: Results are shown in Figure 3.

*Respiratory events*: IED density in sleep with concomitant apnea or hypopnea (3.6 [0.2–8.0] was frequently lower than in sleep without 6.3 [3.7–19.7]), however the difference was not statistically significant (*p* = 0.055) (Figure 3A). When considering a few additional seconds before (+5) and after (+10) the events, the reduction of IED density during RE (2.6 [0.3–6.6]) became significant (sleep without apnea or hypopnea: 6.7 [3.9–20.5], *p* = 0.017). (Figure 3B).

*Apnea alone*: IED in sleep during apnea (0 [0–0]) was significantly lower than in sleep outside apnea (6.7 [3.4–18.0]), *p* < 0.001 (Figure 3C). Results were similar with the extended starting and stopping time (IED in sleep during apnea: 0 [0–0]; outside apnea: 6.4 [3.4–18.1], *p* < 0.001) (Figure 3D).

*Desaturation*: IED density in sleep during desaturation (2.2 [0–7.4]) was significantly lower than in sleep without (6.4 [3.4–18.4]), *p* = 0.009. (Figure 3E). The same was observed with the extended definition (2.6 [0–6.7] vs. 6.8 [3.4–18.5], *p* = 0.012) (Figure 3F).

All *p* values < 0.05 presented above remained significant after control for false discovery (rate of 5%).

*Sleep stage specific analysis*: We performed the same analysis specifically for sleep stages N2, N3 and REM (Figures S1–S3). The results were qualitatively similar, showing in each sleep stage as general trend a reduced IED density during RE, apnea, and desaturation.

### 3.4 IED and Arousal

**FIGURE 4:**
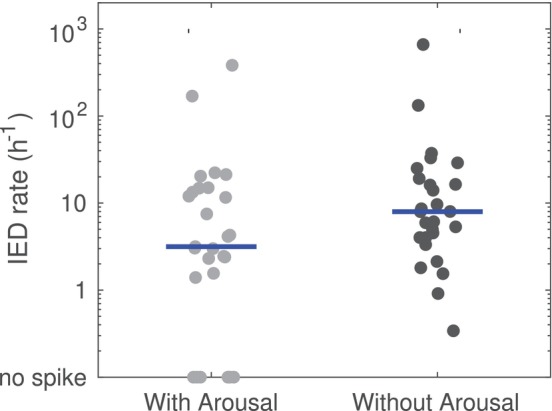
Density of interictal epileptiform discharges (IED) in epochs with and without arousal. IED during N2, N3 and REM sleep was tendentially lower in 30 s scoring epochs containing one or more arousal than in epoch without arousal, however the difference did not reach the significance threshold (Horizontal bars represent the median.

Finally, we investigated the effect of sleep fragmentation on IED. To this end, we compared sleep epochs with arousal and sleep epochs without arousal. We considered only N2, N3 and REM sleep, since epochs scored as N1 can contain wakefulness not scored as arousal (in the wake‐to‐sleep transition). We found a trend towards lower IED density in sleep epochs with arousal (3.2 [1.4–14.4]) than without (8.0 [4.0–18.5]), however the difference was not statistically significant (*p* = 0.090) (Figure 4).

### 3.5 Seizures

Only one patient had epileptic seizures during the PSG recordings: a 42‐year‐old male with structural epilepsy due to a brain tumour had 202 episodes of focal rhythmic activity fulfilling the criteria for electrographical seizures (Hirsch et al. 2021). The density of seizures starting during respiratory events was 6.7/h versus 15.9/h in sleep without RE; 2.9/h during apnea versus 15.9/h outside; 15.7/h during desaturations versus 13.4/h outside.

## 4 Discussion

In this work, we compared the density of interictal epileptic discharges found during polysomnographic recordings under different conditions. First, we found that sleep stages influence the IED density, which was higher during N2 and N3 than in Wake and REM. These results have been previously described (Minecan et al. 2002; Rocamora et al. 2013; Ng and Pavlova 2013) and serve here mainly as quality control for our next analysis. The main goal of our study was to investigate IED with respect to respiratory events and hypoxia in OSA. This question is of scientific and clinical importance, as it might be a first step towards explaining the mechanisms whereby OSA influences seizure control in patients with epilepsy (Goyal et al. 2023). Indeed, an increase of IED during respiratory events or desaturation would have been an indirect argument supporting an immediate effect of hypoxia on pathological neural activity. However, our results show that this is probably not the case. In fact, depending on the definition used for respiratory events, we usually found that IED was significantly *reduced* during RE. This effect was also seen when the analysis was performed independently for different sleep stages, indicating that it was not solely due to a shift in sleep stage following apnea. It must be mentioned, however, that the AASM rules for sleep scoring used in the present study are based on 30 s epochs. Variations on a shorter time‐scale, such as cyclic alternating pattern (Terzano and Parrino 1993) or infraslow‐oscillations (Vanhatalo et al. 2004), have not been considered. Instead, as surrogate marker for shorter changes and for sleep fragmentation, we compared IED in 30 s sleep epochs with and without alpha arousal. While the difference was not statistically significant, there was a trend towards lower IED density in epochs with arousal. These results are interesting since arousals have been linked to IED occurrence (Peter‐Derex et al. 2020; Gumusyayla et al. 2016) and sleep fragmentation has also been postulated as contributing factor linking OSA and severe epilepsy (Goyal et al. 2023; Wächter et al. 2020).

At this point, it is important to mention that the temporal relationship between IEDs and epileptic seizures is complex. There is growing evidence for the existence of circadian or multidian excitatory states in many patients, during which both IED and seizures are more frequent (Baud et al. 2018; Proix et al. 2021). On a shorter time scale, however, there seems to be inter‐individual differences as some patients will have an increase, and other a decrease of IEDs immediately preceding seizures (Baud et al. 2018; Karoly et al. 2021; Schroeder et al. 2023; Karoly et al. 2016). Thus, our study cannot be used to conclude that seizures are less likely to occur during RE (even though it was the case in the single patient in our collective with epileptic seizures). In addition, to the best of our knowledge, none of the studies correlating IED with seizure occurrence did explicitly take RE or arousal into account, so that the relationship between IED and seizures during sleep in patients with OSA remains largely unknown.

While keeping the distinction between seizures and IED in mind, it remains interesting that the latter, a clear pathological finding and one of the EEG hallmarks of epilepsy, was not increased by RE or desaturation. There are several possible explanations, ranging from a sampling effect, to the oscillatory nature of instable sleep or even a short term potential protective effect of arousal and desaturation during sleep: First, one must keep in mind that part of the reduction of IED density observed during apnea could be due to the short total time spent during apnea, together with an overall low density of spikes (if an event is rare, it has a higher chance never to occur during a short‐defined period)—even thought this is unlikely to be the main explanation. Second, our results are in line with the work of Terzano and his group on cycling alternating pattern (CAP). We recall that CAP is an EEG motive found in NREM sleep characterised by recurring occurrence of phasic signals (called ‘phase A,’ usually high‐voltage slow waves) from the background (‘phase B’). CAP is considered a marker of sleep instability (Parrino et al. 2012), with phase A being a general type of arousal and phase B a post arousal rebound. It has been shown that RE are more likely to occur during phase B (Terzano et al. 1996), whereas interictal and ictal epileptic activity is more likely to be found in phase A (Manni et al. 2005; Parrino et al. 2000). Thus, the lack of temporal correlation between RE and IED is not surprising at least in CAP sleep, since they appear in different phases. Third, we note that in our patients IED were more frequent in sleep than in wakefulness. Arousals, which can be associated with RE or desaturation, might thus reduce the IED density simply because they reduce the sleep time. Indeed, epochs with arousal had a tendency to contain less spikes, even though the effect was not statistically significant (Figure 4). Similarly, Peter‐Derex et al. (Peter‐Derex et al. 2020) found that while IED might increase just before and at the beginning of arousals, they could decrease during the arousal. Finally, it is possible—though hypothetical at this stage that RE and hypoxia, which seem to be pro‐epileptogenic over longer time scale (Grigg‐Damberger and Foldvary‐Schaefer 2024), are protective on a very short scale. This hypothesis is in line with several animal studies showing that hypoxia can decrease the release of excitatory neurotransmitters like glutamate, alter the activity of ion channels involved in neuronal firing and lead to decrease in the metabolic activity of neurons (Mishra et al. 2020; Damgaard et al. 2023; Burtscher et al. 2021). There is also evidence that hypercapnia may also have antiepileptic effects, leading to increase in extracellular pH, activation of adenosine receptors and enhancing GABA‐ergic inhibition (Mishra et al. 2020). A neuroprotective effects of hypoxia and hypercapnia, as derived from predominantly animal studies, is still compatible with the observed association between OSA and poor seizure control, which would then involve indirect mechanisms (Damgaard et al. 2023; Ma and Wu 2023), for instance cumulative long‐term effects of inflammatory processes over time, or the disruption by OSA of the restorative functions of sleep such as synaptic down‐scaling (Tononi and Cirelli 2020) or cleansing of cerebro‐spinal fluid (Rasmussen et al. 2018). The bidirectional link can also be reinforced by potential side effects of anti‐seizure medication (e.g., weight gain, muscle relaxation and altered respiratory control) and the direct effect of epilepsy on brain regions responsible for breathing control (Mishra et al. 2020).

### 4.1 Strengths and Limitations

The strengths of this study are the following: (a) a meticulous methodological approach to scoring respiratory events and EEG events by a board certified pneumologist and neurologists with additional EEG certification, (b) the patient population reflected the real‐world interdisciplinary practice of our sleep center, which jointly evaluates patients with neurologic and respiratory diseases like epilepsy and sleep apnea, increasing the generalizability of our findings to similar clinical settings.

The main limitation, which has already been mentioned, is the fact that we studied interictal and not ictal activity. The other limitations mainly derive from the retrospective design of the study. First, since all patients were referred for clinical reasons, there is a potential bias in having severe cases of epilepsy (patients for which OSA had to be ruled out to improve seizure control). The severity of epilepsy is also indicated by the fact that 40% of the cohort were taking more than one anti‐seizure medication (ASM). It is known that ASM can influence sleep, relative percentage of sleep stages (Campana et al. 2017; Liguori et al. 2021) and cyclic alternating pattern (Yeh et al. 2022). Also the effect of ASM on sleep apnea and IED cannot be disentangled. In this population, there was a higher proportion of known or postulated sleep related epileptic activity (it was the referral motive in 40% of patients). We also grouped together patients with different types of epilepsy and IED location, whereas the effect of sleep or sleep deprivation on them is not necessarily identical (Nobili et al. 2022; Renzel et al. 2016). The sample size does not indeed permit a robust analysis for different locations of seizure onset.

## 5 Conclusion

In this work, we compared the density of IED during sleep with and without RE or desaturation. Our major finding is that there is no temporal relationship, and thus probably no direct immediate causative relationship between these events. This result is important, as it highlights the necessity to further investigate the delayed pathophysiological link between OSA and epilepsy, weather due to hypoxia or to another mechanism, which may pave the road for future therapeutic approaches.

## Author Contributions

**Christian M. Horvath:** writing – original draft, conceptualization, investigation, formal analysis, validation, methodology, visualization, data curation, software. **Hristina Drangova:** investigation, writing – original draft, conceptualization, formal analysis, methodology, validation, visualization, data curation, software. **Jakub Stefela:** writing – review and editing, investigation. **Carolin Schäfer:** writing – review and editing, investigation. **Frederic Zubler:** investigation, supervision, conceptualization, methodology, writing – original draft, validation, formal analysis, data curation, software, visualization, resources, project administration.

## Conflicts of Interest

The authors declare no conflicts of interest.

## Supporting information


**Data S1.** Supporting Information.

## Data Availability

The data that support the findings of this study are available on request from the corresponding author. The data are not publicly available due to privacy or ethical restrictions.

## References

[jsr70021-bib-0001] Aldrich, M. , and M. Aldrich . 1999. Sleep Medicine. Oxford University Press.

[jsr70021-bib-0002] Baud, M. O. , J. K. Kleen , E. A. Mirro , et al. 2018. “Multi‐Day Rhythms Modulate Seizure Risk in Epilepsy.” Nature Communications 9, no. 1: 88. 10.1038/s41467-017-02577-y. PMC5758806 29311566

[jsr70021-bib-0003] Berry, R. Q. , and A. Abreu . 2020. “The AASM Manual for the Scoring of Sleep and Associated Events: Rules, Terminology and Technical Specifications, Version 2.6.” American Academy of Sleep Medicine: 56–64.

[jsr70021-bib-0004] Burtscher, J. , R. T. Mallet , M. Burtscher , and G. P. Millet . 2021. “Hypoxia and Brain Aging: Neurodegeneration or Neuroprotection?” Ageing Research Reviews 68: 101343. 10.1016/j.arr.2021.101343. 33862277

[jsr70021-bib-0005] Campana, C. , F. Zubler , S. Gibbs , et al. 2017. “Suppression of Interictal Spikes During Phasic Rapid Eye Movement Sleep: A Quantitative Stereo‐Electroencephalography Study.” Journal of Sleep Research (October) 26, no. 5: 606–613. 10.1111/jsr.12533. 28401614

[jsr70021-bib-0006] Damgaard, V. , J. Mariegaard , J. M. Lindhardsen , H. Ehrenreich , and K. W. Miskowiak . 2023. “Neuroprotective Effects of Moderate Hypoxia: A Systematic Review.” Brain Sciences 13, no. 12: 1648. 10.3390/brainsci13121648. 38137096 PMC10741927

[jsr70021-bib-0007] Devinsky, O. , B. Ehrenberg , G. M. Barthlen , H. S. Abramson , and D. Luciano . 1994. “Epilepsy and Sleep Apnea Syndrome.” Neurology (November) 44, no. 11: 2060. 10.1212/wnl.44.11.2060. 7969960

[jsr70021-bib-0008] Fisher, R. S. , W. van Emde Boas , W. Blume , et al. 2005. “Epileptic Seizures and Epilepsy: Definitions Proposed by the International League Against Epilepsy (ILAE) and the International Bureau for Epilepsy (IBE).” Epilepsia 46, no. 4: 470–472. 10.1111/j.0013-9580.2005.66104.x. 15816939

[jsr70021-bib-0009] Gibbs, S. A. , P. Proserpio , M. Terzaghi , et al. 2016. “Sleep‐Related Epileptic Behaviors and Non‐REM‐Related Parasomnias: Insights From Stereo‐EEG.” Sleep Medicine Reviews (February) 25: 4–20. 10.1016/j.smrv.2015.05.002. 26164370

[jsr70021-bib-0010] Gottlieb, D. J. , and N. M. Punjabi . 2020. “Diagnosis and Management of Obstructive Sleep Apnea.” JAMA 323, no. 14: 1389–1400. 10.1001/jama.2020.3514. 32286648

[jsr70021-bib-0011] Goyal, M. , P. Mishra , and H. Jaseja . 2023. “Obstructive Sleep Apnea and Epilepsy: Understanding the Pathophysiology of the Comorbidity.” International Journal of Physiology, Pathophysiology and Pharmacology 15, no. 4: 105–114. 37736503 PMC10509561

[jsr70021-bib-0012] Grigg‐Damberger, M. , and N. Foldvary‐Schaefer . 2024. “Hypoxia Not AHI in Adults With Sleep Apnea Midlife Markedly Increases Risk of Late‐Onset Epilepsy‐Carosella CM Et al Sleep Apnea, Hypoxia, and Late‐Onset Epilepsy: The Atherosclerosis Risk in Communities Study SLEEP‐2023‐0175.R1.” Sleep (June) 47, no. 6: 1–2. 10.1093/sleep/zsad252. 37777197

[jsr70021-bib-0013] Groppe, D. M. , T. P. Urbach , and M. Kutas . 2011. “Mass Univariate Analysis of Event‐Related Brain Potentials/Fields I: A Critical Tutorial Review.” Psychophysiology 48, no. 12: 1711–1725. 10.1111/j.1469-8986.2011.01273.x. 21895683 PMC4060794

[jsr70021-bib-0014] Gumusyayla, S. , A. Erdal , F. I. Tezer , and S. Saygi . 2016. “The Temporal Relation Between Seizure Onset and Arousal‐Awakening in Temporal Lobe Seizures.” Seizure 39: 24–27. 10.1016/j.seizure.2016.05.005. 27235893

[jsr70021-bib-0015] Hirsch, L. J. , M. W. K. Fong , M. Leitinger , et al. 2021. “American Clinical Neurophysiology Society's Standardized Critical Care EEG Terminology: 2021 Version.” Journal of Clinical Neurophysiology 38, no. 1: 1–29. 10.1097/wnp.0000000000000806. 33475321 PMC8135051

[jsr70021-bib-0016] Kane, N. , J. Acharya , S. Benickzy , et al. 2017. “A Revised Glossary of Terms Most Commonly Used by Clinical Electroencephalographers and Updated Proposal for the Report Format of the EEG Findings. Revision 2017.” Clinical Neurophysiology Practice 2: 170–185. 10.1016/j.cnp.2017.07.002. 30214992 PMC6123891

[jsr70021-bib-0017] Karoly, P. J. , D. R. Freestone , R. Boston , et al. 2016. “Interictal Spikes and Epileptic Seizures: Their Relationship and Underlying Rhythmicity.” Brain 139, no. Pt 4: 1066–1078. 10.1093/brain/aww019. 26912639

[jsr70021-bib-0018] Karoly, P. J. , V. R. Rao , N. M. Gregg , et al. 2021. “Cycles in Epilepsy.” Nature Reviews Neurology (May) 17, no. 5: 267–284. 10.1038/s41582-021-00464-1. 33723459

[jsr70021-bib-0019] Latreille, V. , E. K. St Louis , and M. Pavlova . 2018. “Co‐Morbid Sleep Disorders and Epilepsy: A Narrative Review and Case Examples.” Epilepsy Research 145: 185–197. 10.1016/j.eplepsyres.2018.07.005. 30048932

[jsr70021-bib-0020] Liguori, C. , M. Toledo , and S. Kothare . 2021. “Effects of Anti‐Seizure Medications on Sleep Architecture and Daytime Sleepiness in Patients With Epilepsy: A Literature Review.” Sleep Medicine Reviews 60: 101559. 10.1016/j.smrv.2021.101559. 34710770

[jsr70021-bib-0021] Lin, Z. , Q. Si , and Z. Xiaoyi . 2017. “Obstructive Sleep Apnoea in Patients With Epilepsy: A Meta‐Analysis.” Sleep and Breathing 21, no. 2: 263–270. 10.1007/s11325-016-1391-3. 27473532

[jsr70021-bib-0022] Ma, Y. , and Q. Wu . 2023. “Intermittent Hypoxia: Linkage Between OSAS and Epilepsy.” Frontiers in Pharmacology 14: 1230313. 10.3389/fphar.2023.1230313. 38074156 PMC10701596

[jsr70021-bib-0023] Manni, R. , E. Zambrelli , R. Bellazzi , and M. Terzaghi . 2005. “The Relationship Between Focal Seizures and Sleep: An Analysis of the Cyclic Alternating Pattern.” Epilepsy Research 67, no. 1–2: 73–80. 10.1016/j.eplepsyres.2005.08.008. 16185848

[jsr70021-bib-0024] Nguyen‐Michel, V. H. , O. Pallanca , V. Navarro , S. Dupont , M. Baulac , and C. Adam . 2015. “How Are Epileptic Events Linked to Obstructive Sleep Apneas in Epilepsy?” Seizure 24: 121–123. 10.1016/j.seizure.2014.09.004. 25288133

[jsr70021-bib-0025] Minecan, D. , A. Natarajan , M. Marzec , and B. Malow . 2002. “Relationship of Epileptic Seizures to Sleep Stage and Sleep Depth.” Sleep (December) 25, no. 8: 899–904. 12489898

[jsr70021-bib-0026] Mishra, P. , H. Jaseja , and M. Goyal . 2020. “A Critical Analysis of the Purported Role of Hypoxaemia in the Comorbidity of Obstructive Sleep Apnoea and Epilepsy.” Clinical Physiology and Functional Imaging (January) 41, no. 1: 4–9. 10.1111/cpf.12672. 33068455

[jsr70021-bib-0027] Nadkarni, M. A. , D. Friedman , and O. Devinsky . 2012. “Central Apnea at Complex Partial Seizure Onset.” Seizure 21, no. 7: 555–558. 10.1016/j.seizure.2012.04.001. 22726818

[jsr70021-bib-0028] Ng, M. , and M. Pavlova . 2013. “Why Are Seizures Rare in Rapid Eye Movement Sleep? Review of the Frequency of Seizures in Different Sleep Stages.” Epilepsy Research and Treatment 2013: 932790. 10.1155/2013/932790. 23853720 PMC3703322

[jsr70021-bib-0029] Nishimura, Y. , Y. Saito , N. Kondo , et al. 2015. “Ictal Central Apnea and Bradycardia in Temporal Lobe Epilepsy Complicated by Obstructive Sleep Apnea Syndrome.” Epilepsy and Behavior Case Reports 4: 41–44. 10.1016/j.ebcr.2015.05.001. 26744694 PMC4681874

[jsr70021-bib-0030] Nobili, L. , S. Beniczky , S. H. Eriksson , et al. 2021. “Expert Opinion: Managing Sleep Disturbances in People With Epilepsy.” Epilepsy and Behavior 124: 108341. 10.1016/j.yebeh.2021.108341. 34619543

[jsr70021-bib-0031] Nobili, L. , A. de Weerd , G. Rubboli , et al. 2020. “Standard Procedures for the Diagnostic Pathway of Sleep‐Related Epilepsies and Comorbid Sleep Disorders: A European Academy of Neurology, European Sleep Research Society and International League Against Epilepsy‐Europe Consensus Review.” Journal of Sleep Research 29, no. 6: e13184. 10.1111/jsr.13184. 32959468

[jsr70021-bib-0032] Nobili, L. , B. Frauscher , S. Eriksson , et al. 2022. “Sleep and Epilepsy: A Snapshot of Knowledge and Future Research Lines.” Journal of Sleep Research 31, no. 4: 13622. 10.1111/jsr.13622. PMC9540671 35487880

[jsr70021-bib-0033] Parrino, L. , R. Ferri , O. Bruni , and M. G. Terzano . 2012. “Cyclic Alternating Pattern (CAP): The Marker of Sleep Instability.” Sleep Medicine Reviews 16, no. 1: 27–45. 10.1016/j.smrv.2011.02.003. 21616693

[jsr70021-bib-0034] Parrino, L. , A. Smerieri , M. C. Spaggiari , and M. G. Terzano . 2000. “Cyclic Alternating Pattern (CAP) and Epilepsy During Sleep: How a Physiological Rhythm Modulates a Pathological Event.” Clinical Neurophysiology 111, no. 2: S39–S46. 10.1016/s1388-2457(00)00400-4. 10996553

[jsr70021-bib-0035] Peter‐Derex, L. , P. Klimes , V. Latreille , S. Bouhadoun , F. Dubeau , and B. Frauscher . 2020. “Sleep Disruption in Epilepsy: Ictal and Interictal Epileptic Activity Matter.” Annals of Neurology 88, no. 5: 907–920. 10.1002/ana.25884. 32833279

[jsr70021-bib-0036] Proix, T. , W. Truccolo , M. G. Leguia , et al. 2021. “Forecasting Seizure Risk in Adults With Focal Epilepsy: A Development and Validation Study.” Lancet Neurology 20, no. 2: 127–135. 10.1016/s1474-4422(20)30396-3. 33341149 PMC7968722

[jsr70021-bib-0037] Rasmussen, M. K. , H. Mestre , and M. Nedergaard . 2018. “The Glymphatic Pathway in Neurological Disorders.” Lancet Neurology 17, no. 11: 1016–1024. 10.1016/s1474-4422(18)30318-1. 30353860 PMC6261373

[jsr70021-bib-0038] Renzel, R. , C. R. Baumann , and R. Poryazova . 2016. “EEG After Sleep Deprivation Is a Sensitive Tool in the First Diagnosis of Idiopathic Generalized but Not Focal Epilepsy.” Clinical Neurophysiology 127, no. 1: 209–213. 10.1016/j.clinph.2015.06.012. 26118491

[jsr70021-bib-0039] Rocamora, R. , R. G. Andrzejak , J. Jiménez‐Conde , and C. E. Elger . 2013. “Sleep Modulation of Epileptic Activity in Mesial and Neocortical Temporal Lobe Epilepsy: A Study With Depth and Subdural Electrodes.” Epilepsy and Behavior 28, no. 2: 185–190. 10.1016/j.yebeh.2013.04.010. 23751358

[jsr70021-bib-0040] Scheffer, I. E. , S. Berkovic , G. Capovilla , et al. 2017. “ILAE Classification of the Epilepsies: Position Paper of the ILAE Commission for Classification and Terminology.” Epilepsia 58, no. 4: 512–521. 10.1111/epi.13709. 28276062 PMC5386840

[jsr70021-bib-0041] Schroeder, G. M. , P. J. Karoly , M. Maturana , et al. 2023. “Chronic Intracranial EEG Recordings and Interictal Spike Rate Reveal Multiscale Temporal Modulations in Seizure States.” Brain Commun 5, no. 5: fcad205. 10.1093/braincomms/fcad205. 37693811 PMC10484289

[jsr70021-bib-0042] Somboon, T. , M. M. Grigg‐Damberger , and N. Foldvary‐Schaefer . 2019. “Epilepsy and Sleep‐Related Breathing Disturbances.” Chest 156, no. 1: 172–181. 10.1016/j.chest.2019.01.016. 30711481

[jsr70021-bib-0043] Souques, A. 1936. Etapes de la Neurologie Dans L'antiquite Grecque. Masson.

[jsr70021-bib-0044] Terzano, M. G. , and L. Parrino . 1993. “Clinical Applications of Cyclic Alternating Pattern.” Physiology and Behavior 54, no. 4: 807–813. 10.1016/0031-9384(93)90096-x. 8248361

[jsr70021-bib-0045] Terzano, M. G. , L. Parrino , M. Boselli , M. C. Spaggiari , and G. Di Giovanni . 1996. “Polysomnographic Analysis of Arousal Responses in Obstructive Sleep Apnea Syndrome by Means of the Cyclic Alternating Pattern.” Journal of Clinical Neurophysiology 13, no. 2: 145–155. 10.1097/00004691-199603000-00005. 8849969

[jsr70021-bib-0046] Tononi, G. , and C. Cirelli . 2020. “Sleep and Synaptic Down‐Selection.” European Journal of Neuroscience 51, no. 1: 413–421. 10.1111/ejn.14335. 30614089 PMC6612535

[jsr70021-bib-0047] Vanhatalo, S. , J. M. Palva , M. D. Holmes , J. W. Miller , J. Voipio , and K. Kaila . 2004. “Infraslow Oscillations Modulate Excitability and Interictal Epileptic Activity in the Human Cortex During Sleep.” Proceedings of the National Academy of Sciences 101, no. 14: 5053–5057. 10.1073/pnas.0305375101. PMC387372 15044698

[jsr70021-bib-0048] Wächter, M. , J. W. Kantelhardt , M. R. Bonsignore , et al. 2020. “Unique Sleep‐Stage Transitions Determined by Obstructive Sleep Apnea Severity, Age and Gender.” Journal of Sleep Research (April) 29, no. 2: e12895. 10.1111/jsr.12895. 31347213

[jsr70021-bib-0049] Yeh, W. C. , H. J. Lin , Y. S. Li , et al. 2022. “Non‐Rapid Eye Movement Sleep Instability in Adults With Epilepsy: A Systematic Review and Meta‐Analysis of Cyclic Alternating Pattern.” Sleep (April) 45, no. 4: 4–8. 10.1093/sleep/zsac041. 35192721

[jsr70021-bib-0050] Zitting, K. M. , B. J. Lockyer , A. Azarbarzin , et al. 2023. “Association of Cortical Arousals With Sleep‐Disordered Breathing Events.” Journal of Clinical Sleep Medicine (May) 19, no. 5: 899–912. 10.5664/jcsm.10492. 36708264 PMC10152355

