# Supplementary material for: Refuting a Temporal Correlation: Interictal Epileptic Discharges Do Not Preferentially Occur During Respiratory Events in Patients With Sleep‐Related Breathing Disorder and Epilepsy
Source: J Sleep Res. 2025 Feb 23;34(6):e70021. doi: 10.1111/jsr.70021 (PMC12592808; doi:10.1111/jsr.70021)
Supplement: Supplementary file 1 — Data S1. Supporting Information. [file JSR-34-e70021-s001.docx]

**Refuting a temporal correlation: interictal epileptic discharges do not preferentially occur during respiratory events in patients with sleep-related breathing disorder and epilepsy**

Christian M. Horvath^1*^, Hristina Drangova^2*^, Jakub Stefela^2,3^, Carolin Schäfer^2^, Frederic Zubler^2,4^

1. Department of Pulmonary Medicine, Allergology and Clinical

Immunology, Bern University Hospital, University of Bern, Bern, Switzerland

2. Sleep-Wake-Epilepsy-Center, Department of Neurology, Inselspital, Bern University Hospital, University of Bern, Bern, Switzerland

3. Department of Neurology, St. Anne's University Hospital and Faculty of Medicine, Masaryk University, Brno, Czech Republic

4. Department of Neurology, Spitalzentrum Biel, University of Bern, Biel/Bienne, Switzerland

*These two authors contributed equally to this work

**Corresponding Author**

Hristina Drangova, MD

Department of Neurology

Inselspital Bern

Freiburgstrasse 20, 3010 Bern

Switzerland

E-Mail: drangova@outlook.com

Table S1: Polysomnographic details

| Patient | Biological sex | Age (years) | Indication for PSG | 10/20-EEG montage | Sleep apnea type | Total AHI [h^-1^] | Central AI [h^-1^] | Obstructive AI [h^-1^] | REM AHI [h^-1^] | NREM AHI [h^-1^] |
| --- | --- | --- | --- | --- | --- | --- | --- | --- | --- | --- |
| 1 | m | 51 | SDB | yes | obstructive | 13.2 | 0 | 0.3 | 7.1 | 13.5 |
| 2 | m | 66 | SDB | yes | obstructive | 25.1 | 0.9 | 7.8 | 72.7 | 21.9 |
| 3 | m | 55 | SDB | no | obstructive | 14.6 | 0.5 | 4.2 | 27.6 | 12.5 |
| 4 | f | 61 | SDB; SREA | yes | obstructive | 95.1 | 0 | 34.8 | 86.7 | 95.6 |
| 5 | m | 49 | SDB; SREA | yes | obstructive | 20.4 | 0 | 1.6 | 13.9 | 21.1 |
| 6 | m | 52 | SDB | yes | obstructive | 17.1 | 0 | 0.6 | 18.1 | 16.7 |
| 7 | m | 42 | SDB | no | obstructive | 64.3 | 13.8 | 14.1 | 1.3 | 72.1 |
| 8 | f | 22 | SREA | yes | obstructive | 10 | 0 | 0 | 0 | 10 |
| 9 | f | 34 | SDB | no | obstructive | 14.8 | 0 | 0 | 14.4 | 14.9 |
| 10 | m | 62 | SDB; SREA | yes | obstructive | 15.9 | 1.2 | 2.1 | 16.2 | 15.9 |
| 11 | m | 51 | SDB; SREA | yes | obstructive | 21.3 | 0.3 | 8.9 | 11.6 | 22 |
| 12 | m | 33 | SDB; SREA | yes | obstructive | 17 | 1.4 | 1.4 | 21.8 | 16.8 |
| 13 | f | 49 | SDB; SREA | yes | obstructive | 14.2 | 0.4 | 0.2 | 18.2 | 13.3 |
| 14 | m | 30 | SREA | yes | obstructive | 11.1 | 0.4 | 0 | 28 | 7.9 |
| 15 | m | 28 | SDB | yes | obstructive | 94 | 2.2 | 69.7 | 73.8 | 96.4 |
| 16 | m | 59 | SDB | yes | obstructive | 24.9 | 0.4 | 0.4 | 14.1 | 26.3 |
| 17 | m | 77 | SDB | yes | obstructive | 24.2 | 0.6 | 12.9 | 43.4 | 20.5 |
| 18 | f | 47 | SREA | yes | obstructive | 28.6 | 0 | 1 | 0 | 28.6 |
| 19 | f | 74 | SREA | yes | obstructive | 10 | 0.4 | 2.9 | 12.5 | 8.1 |
| 20 | m | 38 | SDB | no | obstructive | 11.8 | 0.5 | 2.6 | 11.4 | 12 |
| 21 | f | 49 | SDB | yes | obstructive | 13.9 | 0.4 | 0.2 | 26.4 | 12.7 |
| 22 | f | 67 | SDB | yes | obstructive | 20.5 | 0 | 3.7 | 50.5 | 12.2 |
| 23 | f | 33 | EDS | yes | obstructive | 11.5 | 0.4 | 0 | 3.2 | 12 |
| 24 | m | 42 | SDB | yes | obstructive | 47.9 | 4.3 | 17 | 73.1 | 44.9 |
| 25 | m | 55 | SDB | yes | obstructive | 16.7 | 0 | 2.1 | 12.3 | 17.7 |
| 26 | m | 47 | SREA | yes | obstructive | 28.8 | 0.8 | 0.8 | 8.6 | 33.3 |
| 27 | m | 57 | SDB | yes | obstructive | 25.1 | 0.2 | 2.3 | 24 | 25.2 |

Abbreviations: PSG (polysomnography), m (male), f (female), AHI (apnea-hypopnea index), SDB (sleep disordered breathing), SREA (suspected sleep related epileptic activity), EDS (excessive daytime sleepiness), AI (apnea index), REM (rapid eye movement)

Table S2: Detailed sleep stages

| Patient | WASO (min) | TST (min) | Sleep Onset (min) | SE (%) | N1 (min) | N1 (% TST) | N2 (min) | N2 (% TST) | N3 (min) | N3 (% TST) | REM (min) | REM (% TST) | Wake (min) |
| --- | --- | --- | --- | --- | --- | --- | --- | --- | --- | --- | --- | --- | --- |
| 1 | 58.5 | 218 | 12.5 | 75.4 | 38 | 17.4 | 94 | 43.1 | 77.5 | 35.5 | 8.5 | 3.9 | 71 |
| 2 | 97 | 268 | 10 | 71.5 | 85.5 | 31.9 | 138 | 51.5 | 28 | 10.4 | 16.5 | 6.2 | 107 |
| 3 | 54.5 | 369.5 | 5 | 86.1 | 93.5 | 25.3 | 197.5 | 53.5 | 21 | 5.7 | 57.5 | 15.6 | 59.5 |
| 4 | 103 | 169 | 66 | 50 | 75 | 44.4 | 85 | 50.3 | 0 | 0 | 9 | 5.3 | 169 |
| 5 | 39.5 | 347 | 4.5 | 88.7 | 110 | 31.7 | 123 | 35.4 | 79.5 | 22.9 | 34.5 | 9.9 | 44 |
| 6 | 30.5 | 281 | 11.5 | 87 | 35 | 12.5 | 134.5 | 47.9 | 42 | 14.9 | 69.5 | 24.7 | 42 |
| 7 | 48.5 | 409 | 1.5 | 89.1 | 191.5 | 46.8 | 166 | 40.6 | 6 | 1.5 | 45.5 | 11.1 | 50 |
| 8 | 186.5 | 165.5 | 25.1 | 43.9 | 122.5 | 74 | 43 | 26 | 0 | 0 | 0 | 0 | 211.6 |
| 9 | 154 | 299.5 | 11.5 | 64.4 | 57.5 | 19.2 | 98.5 | 32.9 | 85 | 28.4 | 58.5 | 19.5 | 165.5 |
| 10 | 18.5 | 313 | 0.5 | 94.3 | 55 | 17.6 | 190.5 | 60.9 | 15.5 | 4.9 | 52 | 16.6 | 19 |
| 11 | 91 | 223 | 11 | 68.6 | 98.5 | 44.2 | 75 | 33.6 | 34 | 15.2 | 15.5 | 7 | 102 |
| 12 | 122 | 173 | 56 | 49.3 | 52 | 30.1 | 88.5 | 51.2 | 27 | 15.6 | 5.5 | 3.2 | 178 |
| 13 | 12 | 333 | 14 | 92.8 | 33.5 | 10.1 | 140.5 | 42.2 | 93 | 27.9 | 66 | 19.8 | 26 |
| 14 | 18 | 319 | 30 | 86.9 | 64 | 20.1 | 146.5 | 45.9 | 57 | 17.9 | 51.5 | 16.1 | 48 |
| 15 | 186.5 | 189.5 | 13 | 48.7 | 138 | 72.8 | 32 | 16.9 | 0 | 0 | 19.5 | 10.3 | 199.5 |
| 16 | 55.5 | 296 | 6.5 | 82.7 | 93 | 31.4 | 111.5 | 37.7 | 57.5 | 19.4 | 34 | 11.5 | 62 |
| 17 | 68.5 | 324.5 | 2 | 82.2 | 65.5 | 20.2 | 197 | 60.7 | 9.5 | 2.9 | 52.5 | 16.2 | 70.5 |
| 18 | 131 | 126 | 155 | 30.6 | 29 | 23 | 62.5 | 49.6 | 34.5 | 27.4 | 0 | 0 | 286 |
| 19 | 44.5 | 326 | 5.5 | 86.7 | 33 | 10.1 | 84 | 25.8 | 75 | 23 | 134 | 41.1 | 50 |
| 20 | 27.5 | 340 | 4.5 | 91.4 | 52.5 | 15.4 | 163 | 47.9 | 50.5 | 14.8 | 74 | 21.8 | 32 |
| 21 | 72.5 | 276 | 5.5 | 78 | 66 | 23.9 | 172 | 62.3 | 13 | 4.7 | 25 | 9.1 | 78 |
| 22 | 88 | 263 | 37 | 67.8 | 128.5 | 48.9 | 58 | 22.1 | 19.5 | 7.4 | 57 | 21.7 | 125 |
| 23 | 196 | 298.5 | 11.5 | 59 | 84 | 28.1 | 119.5 | 40 | 76 | 25.5 | 19 | 6.4 | 207.5 |
| 24 | 65 | 307 | 0 | 82.5 | 124.5 | 85.5 | 85.5 | 27.9 | 65 | 27.9 | 32 | 10.4 | 65 |
| 25 | 34.5 | 342 | 12.5 | 87.9 | 78.5 | 23 | 135.5 | 39.6 | 64.5 | 18.9 | 64.5 | 18.9 | 47 |
| 26 | 27.5 | 304 | 2.5 | 91 | 84 | 27.6 | 136.5 | 44.9 | 28 | 9.2 | 55.5 | 18.3 | 30 |
| 27 | 14 | 335 | 2 | 95.4 | 30.5 | 9.1 | 231.5 | 69.1 | 28 | 8.4 | 45 | 13.4 | 16 |

Data are given as median with quartile range 1 to 3.

Abbreviations: WASO (wake after sleep onset), TST (total sleep time), SE (sleep efficiency), N1 to N3 (sleep stages), REM (rapid eye movement)

Table S3: Detailed information on epilepsy

| Patient | Diagnose | Known epilepsy | Type of epileptic activity detected in PSG | Number of spikes | Seizures recorded | IED Density (h^-1^) | Anti-epileptic drugs | Seizure control |
| --- | --- | --- | --- | --- | --- | --- | --- | --- |
| 1 | Structural epilepsy due to traumatic brain injury | Yes | Focal sharp waves | 31 | No | 6.4 | Valproate | Unknown (external patient) |
| 2 | Structural epilepsy due to ischemic stroke | Yes | Focal sharp waves | 1 | No | 0.2 | Levetiracetam | Seizure-free for the past 12 months |
| 3 | Sleep-related hypermotor epilepsy | Yes | Focal spikes | 7 | No | 1.0 | Levetiracetam | Approximately 4 seizures per year |
| 4 | Focal epilepsy of unknown origin | Yes | Focal sharp slow waves, generalized poly spikes | 125 | No | 22.2 | Lamotrigine | Unknown (external patient) |
| 5 | Epilepsy with sleep-related seizures | Yes | Focal spike waves | 5 | No | 0.8 | Oxcarbazepine | Unknown (external patient) |
| 6 | Epilepsy with sleep-related seizures since childhood | Yes | Focal sharp slow waves | 15 | No | 2.8 | Valproate | Seizure-free since 1 month |
| 7 | Structural epilepsy due to a brain tumor | Yes | Focal sharp waves | 1335 | Yes; electrographical and electroclincal seizure | 174.5 | Lamotrigine | Self-reported seizure-free since many years |
| 8 | Focal epilepsy (first diagnosis based on PSG) | No | Focal spikes | 23 | No | 3.8 | None | Occasional focal seizures |
| 9 | Genetic epilepsy (juvenile myoclonus epilepsy) | Yes | Generalized spike waves | 21 | No | 2.7 | Lamotrigine, Zonesamide | 1x provoked bilateral tonic-clonic seizure in the year prior to PSG |
| 10 | Structural epilepsy due to a brain tumor | Yes | Focal spikes | 29 | No | 5.2 | Levetiracetam, Lamotrigine | Frequent sleep-related seizures |
| 11 | Structural epilepsy due to a brain tumor | Yes | Focal spikes | 1764 | No | 326.2 | Phenytoin, Lamotrigine, Phenobarbital | Seizure-free since 7 years |
| 12 | Structural epilepsy due to a focal cortical dysplasia | Yes | Focal spike waves,Focal sharp waves | 30 | No | 5.1 | Lacosamide, Perampanel | 1x sleep-related seizure per 1-2 months |
| 13 | Focal epilepsy of unknown origin | Yes | Focal spike waves, focal sharp waves | 30 | No | 5.0 | Lamotrigine, Levetiracetam | Seizure-free for the past 12 months |
| 14 | Focal epilepsy of unknown origin | Yes | Focal spikes | 110 | No | 18.0 | Lamotrigine | Approximately 1 seizure per year |
| 15 | Focal cortical dysplasia (dysmorphy syndrome) | No | Focal spikes, focal spike waves | 18 | No | 2.8 | None | No clinical seizures |
| 16 | Focal epilepsy of unknown origin | Yes | Focal spike waves | 92 | No | 15.4 | Lamotrigine | Sleep-related and mostly provoked seizures ( |
| 17 | Structural epilepsy after limbic encephalitis | Yes | Focal sharp slow waves | 45 | No | 6.8 | Oxcarbazepine, Lamotrigine, Topiramate | Therapy-resistent epilepsy, 8-13  seizures per month |
| 18 | Structural epilepsy due to focal cortical dysplasia (dysmorphy syndrome) | Yes | Focal sharp waves | 4 | No | 0.6 | Levetiracetam, Lamotrigine | 1-2x sleep-related provoked and unprovoked seizures per year |
| 19 | Structural epilepsy due to hippocampal sclerosis | Yes | Focal spikes | 74 | No | 11.8 | Lamotrigine | Seizure-free for the past 15 months |
| 20 | Structural epilepsy due to arteriovenous malformation | Yes | Focal spike waves | 86 | No | 13.9 | Lamotrigine | Approximately 1 focal seizure per month |
| 21 | Structural epilepsy due to traumatic brain injury | Yes | Focal spike waves | 123 | No | 20.9 | Lamotrigine | Seizure-free for the past 11 years |
| 22 | Structural epilepsy due to brain tumor | Yes | Focal spikes, focal spike waves | 26 | No | 4.0 | Levetiracetam, Pregabalin | Seizure-free for the past 5 years |
| 23 | Focal cortical dysplasia | No | Focal sharp slow waves | 70 | No | 8.3 | None | No clinical seizures |
| 24 | Structural epilepsy due to traumatic brain injury | Yes | Focal sharp slow waves | 15 | No | 2.4 | Lamotrigine, Pregabalin | Seizure-free for the past 6 months |
| 25 | Structural epilepsy due to traumatic brain injury | Yes | Focal sharp slow waves | 41 | No | 6.3 | None | Seizure-free for the past 18 years |
| 26 | Structural epilepsy due to traumatic brain injury | Yes | Focal sharp waves | 100 | No | 18.0 | Lamotrigine, Valproate, Perampanel | Therapy-resistent epilepsy, 3-4 seizures per month |
| 27 | Mesial temporal lobe epilepsy | Yes | Focal sharp slow waves | 9 | No | 1.5 | Levetiracetam, Perampanel, Topiramate | Therapy-resistent epilepsy, 2-3 seizures per month |

**Figure S1:** **Density of interictal epileptiform discharges (IED) during and outside respiratory events in sleep stage N2**

**
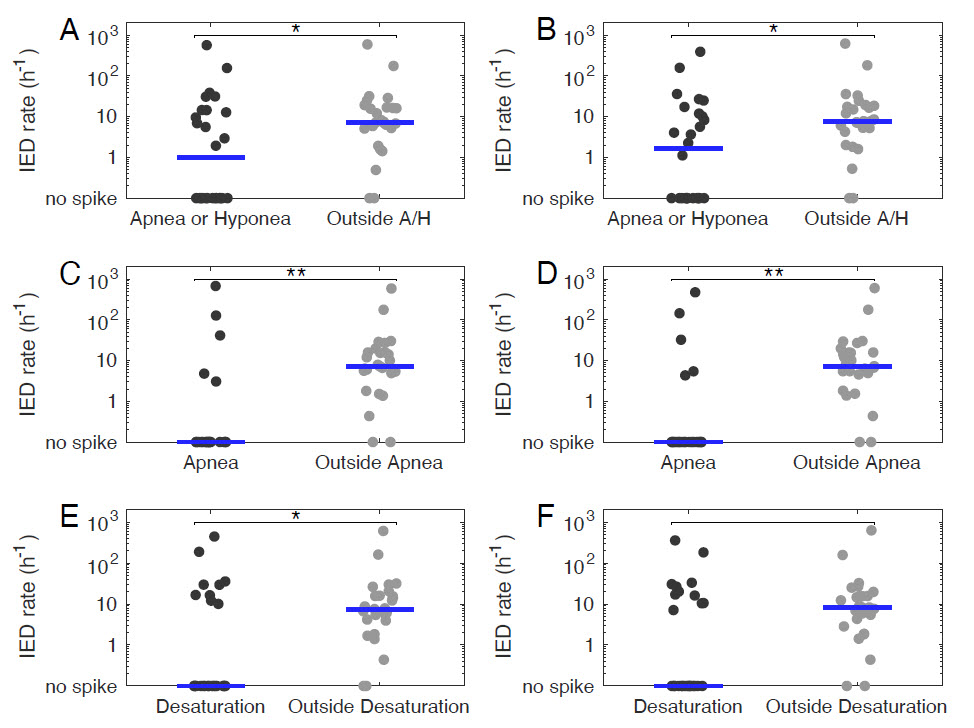
**

**(A**) The IED density was significantly lower in N2 sleep during a respiratory event (RE), that is apnea or a hypopnea (0.97 [0; 14.5]) than in N2 sleep outside an event (7.2 [5.1; 16.7]), p = 0.039.

**(B**) The same applied during using an extended definition (that is, starting 5 second and stopping 10 second after the limits according to the AASM scoring rules): 1.7 [0-11.9] vs 7.5 [4.0-15.9], p = 0.03.

**(C)** The IED was significantly lower during apnea 0 [0-0.8] than outside apnea 7.2 [5.0; 15.8], p = 0.0001

**(D)** The same result was found with the extended definition of apnea 0 [0-0] vs 7.3 [4.7; 15.8], p < 0.0001

**(E)** The IED density was significantly lower in N2 sleep during a desaturation 7.5323 [4.0099; 15.9101] than in N2 sleep during desaturation 0 [0-16.4], p=0.013.

**(F)** The result was similar but did not reach the level for statistical significance for the extended definition of desaturation 0 [0-17.1] vs 8.2 [3.2; 15.7], p=0.06, (horizontal bars represent the median. * p<0.05, ** p < 0.01).

All data are given and median with quartile range (Q1-Q3)

**Figure S2:** **Density of interictal epileptiform discharges (IED) during and outside respiratory events in sleep stage N3**. **Of note, 17 patients had RE, 3 had apnea, and 15 had desaturation in N3**


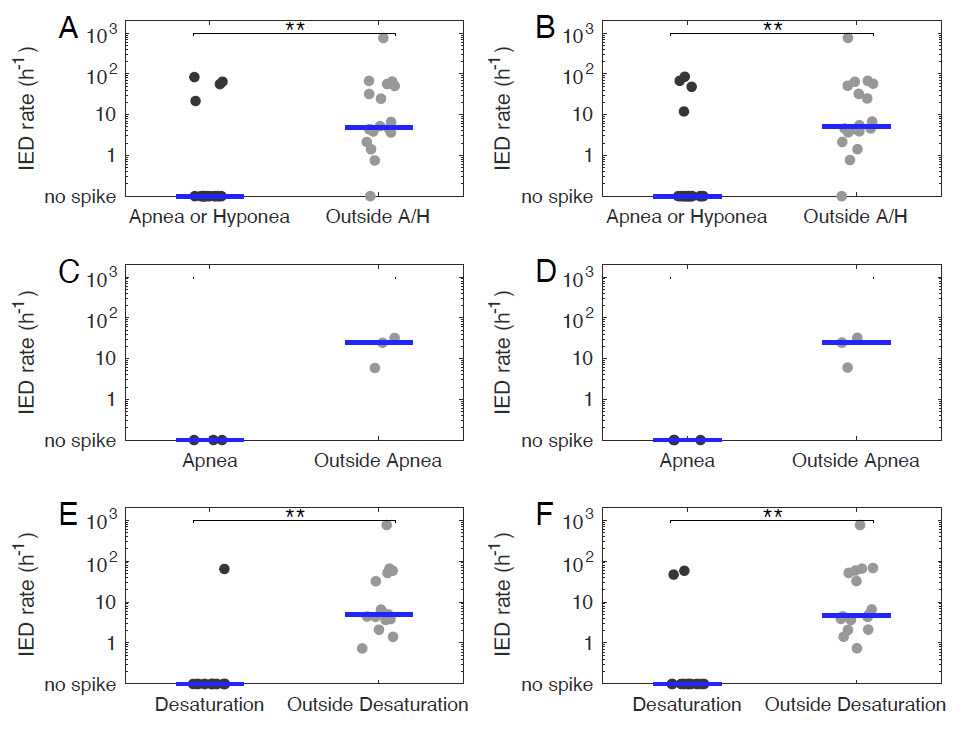


**(A**) The IED density was significantly lower in N3 sleep during a respiratory event (RE), that is apnea or a hypopnea (0 [0-0]) than in sleep outside an event (4.8 [3.7-50.8]), p = 0.0014. **(B**) The same applied during using an extended definition (that is, starting 5 second and stopping 10 second after the limits according to the AASM scoring rules): 0 [0-0] vs 5.0 [3.7-51.4], p = 0.002.

**(C)** The IED was lower during apnea 0 [0-0.0] than outside apnea 24.5 [10.1-30.2], however the difference was not statistically significant due to the low number of patients (p = 0.10) **(D)** The same result was found with the extended definition of apnea 0 [0-0] vs 24.6 [10.7-30.5], p =0.10 **(E)** The IED density was significantly lower in N3 sleep during a desaturation 0 [0-0] than in sleep without desaturation 5.0 [3.7-46.6], p<0.001.

**(F)** The result was similar for the extended definition of desaturation 0 [0-0] vs 4.7 [2.9-55], p<0.001. (Horizontal bars represent the median, * p<0.05, ** p < 0.01).

All data are given and median with quartile range (Q1-Q3)

**Figure S3:** **Density of interictal epileptiform discharges (IED) during and outside respiratory events during REM sleep**. **Of note, 25 patients had RE, 17 had apnea, and 22 had desaturations in N3**.


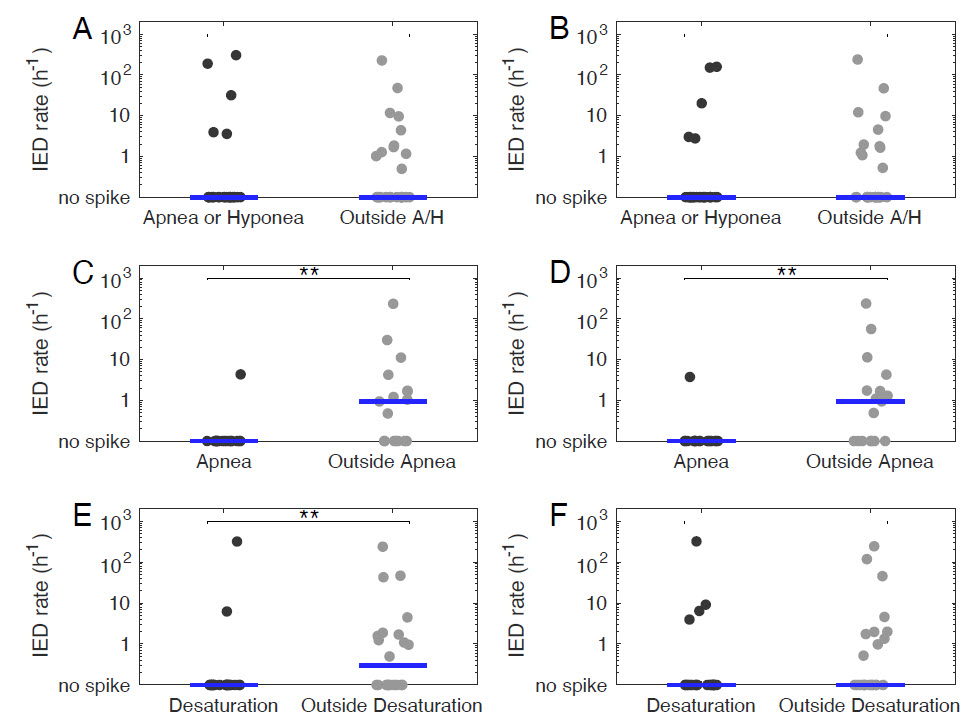


**(A**) The IED density was lower in REM sleep during a respiratory event (RE), that is apnea or a hypopnea, (0 [0,0]) than in sleep outside an event (0 [0-1.7]) without reaching the statistical significance p = 0.16.

**(B**) The same applied during using an extended definition (that is, starting 5 second and stopping 10 second after the limits according to the AASM scoring rules): 0 [0-0] vs 0 [0-1.8], p = 0.15.

**(C)** The IED was significantly lower during apnea 0 [0-0] than outside apnea 0.9 [0-2.3], p = 0.002

**(D)** The same result was found with the extended definition of apnea 0 [0-0] vs 0.9 [0-2.4,15.8], p = 0.002

**(E)** The IED density was significantly lower in REM sleep during a desaturation 0 [0,0] than in sleep during desaturation 0.24 [0-1.7], p=0.008. **(F)** The result was similar but did not reach the level for statistical significance for the extended definition of desaturation 0 [0-0] vs 0 [0-2.0], p=0.06,

(Horizontal bars represent the median. * p<0.05, ** p < 0.01).

All data are given and median with quartile range (Q1-Q3)
